# Tone perception in Mandarin-speaking school age children with otitis media with effusion

**DOI:** 10.1371/journal.pone.0183394

**Published:** 2017-08-22

**Authors:** Ting Cai, Bradley McPherson, Caiwei Li, Feng Yang

**Affiliations:** 1 Division of Speech and Hearing Sciences, Faculty of Education, The University of Hong Kong, Hong Kong, China; 2 Department of Otorhinolaryngology, Shenzhen Children’s Hospital, Shenzhen, China; 3 Department of Speech Therapy, Shenzhen Children’s Hospital, Shenzhen, China; University of Palermo, ITALY

## Abstract

**Objectives:**

The present study explored tone perception ability in school age Mandarin-speaking children with otitis media with effusion (OME) in noisy listening environments. The study investigated the interaction effects of noise, tone type, age, and hearing status on monaural tone perception, and assessed the application of a hierarchical clustering algorithm for profiling hearing impairment in children with OME.

**Methods:**

Forty-one children with normal hearing and normal middle ear status and 84 children with OME with or without hearing loss participated in this study. The children with OME were further divided into two subgroups based on their severity and pattern of hearing loss using a hierarchical clustering algorithm. Monaural tone recognition was measured using a picture-identification test format incorporating six sets of monosyllabic words conveying four lexical tones under speech spectrum noise, with the signal-to-noise ratio (SNR) conditions ranging from -9 to -21 dB.

**Results:**

Linear correlation indicated tone recognition thresholds of children with OME were significantly correlated with age and pure tone hearing thresholds at every frequency tested. Children with hearing thresholds less affected by OME performed similarly to their peers with normal hearing. Tone recognition thresholds of children with auditory status more affected by OME were significantly inferior to those of children with normal hearing or with minor hearing loss. Younger children demonstrated poorer tone recognition performance than older children with OME. A mixed design repeated-measure ANCOVA showed significant main effects of listening condition, hearing status, and tone type on tone recognition. Contrast comparisons revealed that tone recognition scores were significantly better under -12 dB SNR than under -15 dB SNR conditions and tone recognition scores were significantly worse under -18 dB SNR than those obtained under -15 dB SNR conditions. Tone 1 was the easiest tone to identify and Tone 3 was the most difficult tone to identify for all participants, when considering -12, -15, and -18 dB SNR as within-subject variables. The interaction effect between hearing status and tone type indicated that children with greater levels of OME-related hearing loss had more impaired tone perception of Tone 1 and Tone 2 compared to their peers with lesser levels of OME-related hearing loss. However, tone perception of Tone 3 and Tone 4 remained similar among all three groups. Tone 2 and Tone 3 were the most perceptually difficult tones for children with or without OME-related hearing loss in all listening conditions.

**Conclusions:**

The hierarchical clustering algorithm demonstrated usefulness in risk stratification for tone perception deficiency in children with OME-related hearing loss. There was marked impairment in tone perception in noise for children with greater levels of OME-related hearing loss. Monaural lexical tone perception in younger children was more vulnerable to noise and OME-related hearing loss than that in older children.

## Introduction

**Fig 1:**
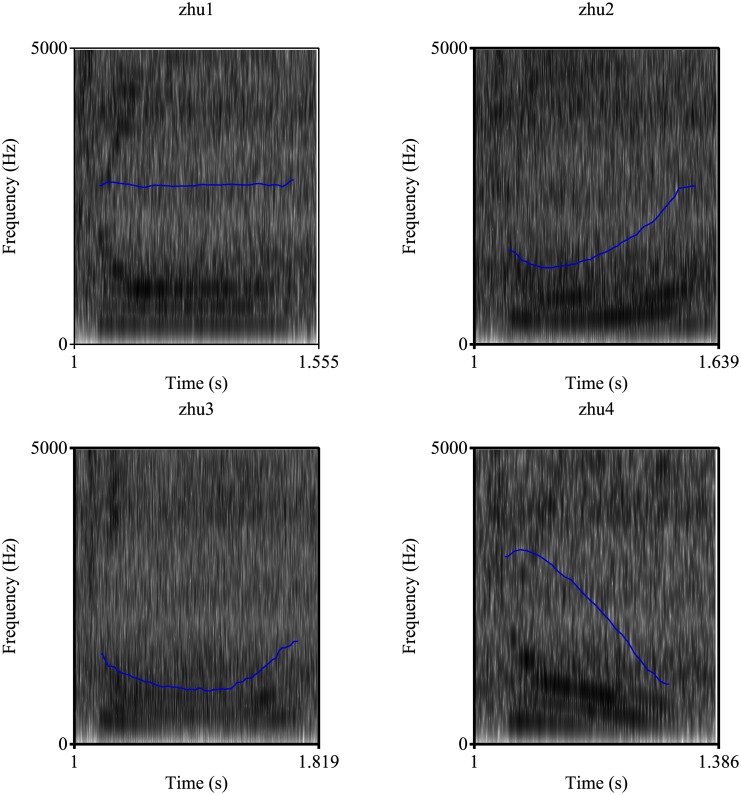
Spectrograms of four Mandarin lexical tone stimuli used in the study.

More than 70% of all the languages in the world are tone languages and approximately one half of the global population speak a tonal language [1]. Tones differ in dimensions of pitch, direction, length, extreme endpoint and slope [2]. Acoustic parameters related with tone primarily include contour and movement of fundamental frequency (F0) [3]. In Mandarin, there are four lexical tones, which can be described as high level (Tone 1), high rising (Tone 2), low dipping (Tone 3) and high falling (Tone 4) based on F0 contours [4]. An example of the spectrograms of the four lexical tones in Mandarin is displayed in Fig 1. Tone recognition is primarily cued by F0 and higher harmonics [3, 5, 6]. Duration, amplitude contour, and spectral envelope may be utilized as secondary cues, especially when F0 is compromised [7–11].

Tones are carried on vowels and denote different meanings for the same monosyllabic word. For example, the syllable /zhu/ may mean “pig”, “bamboo”, “cook”, or “pillar” with different tones. Misperception of lexical tones may hinder word or sentence perception. When F0 variation is manipulated to create a flattened contour, speech perception of Mandarin sentences remains relatively intact in a quiet environment. However, significant reductions in speech perception have been detected under noisy listening conditions. The difference indicates the importance of tone perception on speech understanding in noise [12–15].

Spectrograms of four tones of a monosyllabic word “zhu” are shown as examples. The contours of F0 are denoted by blue lines. The duration is indicated in seconds (s).

Tone perception has been reported to be related to low frequency hearing acuity. Zhang and McPherson [16] found that employing a low-frequency cut, a widely used hearing aid fitting strategy to improve speech intelligibility in noise, impeded the tone recognition ability of normal hearing Mandarin listeners in adverse noise conditions. In other words, when F0 information is filtered, other acoustic cues such as temporal and spectral parameters are sufficient to cue tone perception in a quiet environment. However, those acoustic cues alone cannot maintain adequate tone recognition performance in noisy conditions. Wang et al. [17] analysed the correlation between tone recognition performance and pure tone threshold at frequencies from 250 Hz to 4000 Hz in adults with sensorineural hearing loss. Correlation between these two variables decreased for higher frequencies and the correlation was the strongest at 250 Hz. However, the correlation between tone recognition performance and pure tone threshold at 250 Hz was not significantly higher than those at other frequencies.

Tone perception in prelingually deaf children who wear cochlear implants is reported to be generally poor due to the absence of F0 information [17–20]. Adults and children with sensorineural hearing loss are also reported to have impaired tone perception in quiet and in noisy environments compared to their counterparts with normal hearing [17, 21, 22]. Very little is known about the tone perception abilities of children with hearing loss related to otitis media with effusion (OME), which is the most common middle ear disease during childhood [23]. Conductive hearing loss is the most frequent complication of OME, typically owing to the increased stiffness and mass of the tympanum caused by middle ear effusion [24]. Children with OME have been reported to generally be more affected for low frequency hearing acuity [25]. Therefore it is not unreasonable to raise the question as to whether tone perception is also affected in children with OME-related conductive hearing loss as it is in children with sensorineural hearing loss.

A hierarchical clustering algorithm has been described in a previous study that profiles children with ears diagnosed with OME into groups of different hearing status based on pure tone thresholds [26]. In that study, four clusters were created, based on pure tone configurations. Children categorised into Cluster 1 and Cluster 2 were found to have comparable monaural sentence perception in noise and in quiet with peers with normal hearing (NH), while children categorised into Cluster 3 and Cluster 4 performed significantly poorer than children in Cluster 1 and Cluster 2. Therefore children assigned to Clusters 1 and 2 were considered to be at lower risk of sentence perception impairment and children who assigned to Clusters 3 and 4 were considered to be at higher risk of sentence perception impairment. It remains to be determined whether tone recognition in noise in children with OME also may be stratified using this method. Since this clustering was based on monaural pure tone thresholds and the sentence perception was also evaluated monaurally, lexical tone perception in the present study was assessed monaurally to simplify this initial analysis.

The developmental process of Mandarin lexical tone perception is not well established. There is no consensus regarding the chronological age at which children acquire adult-like tone perception, especially in noisy environments. The phonemic acquisition of Mandarin lexical tones was reported to occur before two years of age in normally developing children [27–29]. Yuen and Yuan reported that tone recognition in noise was stable among children aged four to nine years and not significantly different to that in adults [30]. Similarly, Zhu, Wong, and Chen also found that seven-year-old children could identify more than 90% of lexical tones correctly at -10 dB signal-to-noise ratio (SNR) [31]. However, Mao and Xu reported that children with NH only achieve 77.5% correct under -6 dB SNR condition [20]. General speech perception in noise is considered not to be fully developed until the age of 13 to 15 in children with NH [32]. OME leads to temporary but fluctuant hearing loss which may exert extra challenge in segregating target auditory information from background noise. The interaction of age and hearing impairment associated with OME on tone perception in noise is not clear.

The four Mandarin lexical tones have been noted to have different levels of recognition difficulty, partially due to both their similarities and disparities in acoustic characteristics. For children with NH, Wong et al. [33] found that Tone 3 was the most difficult tone to perceive in quiet for children less than three years old. Zheng [34] reported that the Tone 2/ Tone 3 contrast was the most confused tone pair in quiet for children up to five years of age with NH. For NH children in a noisy listening environment, Zhu et al. [31] indicated that the Tone 1/ Tone 3 contrast was the easiest to discriminate and Mao and Xu. [20] found that tone recognition scores were lowest for Tone 3, and the Tone 2/ Tone 3 contrast was the most confused tone pair. Zhu et al. [31] also investigated tone perception in children with profound hearing loss and revealed that the Tone 1/ Tone 3 contrast yielded the highest score and the Tone 1/ Tone 2 and Tone 2/ Tone 3 contrasts showed poorer discrimination scores than other tone contrasts in a quiet condition. In a noisy environment, there was no significant difference in tone recognition performance among all six tone contrasts. For adult populations with normal hearing and with hearing loss, similar findings have been reported. In a number of studies, adult listeners had more difficulties perceiving Tone 2 and Tone 3 correctly than perceiving Tone 1 and Tone 4, both in quiet and in noisy listening conditions [17, 21, 35]. However, Lee et al. [36] reported that tone recognition performance for Tone 1 and Tone 2 was better than for Tone 3 and Tone 4 for adults with NH in noisy listening conditions. A summary of the main findings on Mandarin tone identification are displayed in Table 1.

**Table 1:** Summary of findings of studies on Mandarin tone identification.

Study	Age range (year)	Sample size	Hearing status	Testing environment	Test material	Test administration	Main finding
Wong 2005	2;10–3;4	13	NH	Quiet	36 monosyllabic word	4AFC picture identification	Hardest tone: Tone 3
Zheng 2009	2–5	92	NH	Quiet	48 monosyllabic word	2AFC picture identification	Most confused tone contrast: Tone 2/Tone 3
Zhu 2014a	7	50	NH	-10 to -30 dB SNR (SSN)	36 monosyllabic words	4AFC picture identification	Least confused tone contrast: Tone 1/Tone 3
Mao 2016	3.41–6.6	52	NH	12 to -6 dB SNR (SSN)	Monosyllabic word	2AFC picture identification	Hardest tone: Tone 3. Most confused tone contrast: Tone 2/Tone 3
Zhu 2014b	5;4–12;6	41	28–51.7 dB HL aided	Quiet	60 monosyllabic words	4AFC picture identification	Most confused tone contrasts: Tone 1/Tone 2 and Tone 2/Tone 3
Zhu 2014b	5;4–12;6	41	28–51.7 dB HL aided	5 to -10 dB SNR (SSN)	60 monosyllabic words	4AFC picture identification	No significant difference among all tone contrasts
Liu 2000	15–50	18	26–70 dB HL unaided	Quiet	96 monosyllabic vowels	Tone repetition and 4AFC tone identification	Tone 2 and Tone 3 are harder than Tone 1 and Tone 4
Wang 2012	11–56	41	41–90 dB HL SNHL unaided	Quiet	64 monosyllabic words	4AFC tone identification	Tone 2 and Tone 3 are harder than Tone 1 and Tone 4. Most confused tone contrast: Tone 2/Tone 3
Krenmayr 2011	21–36	16	NH	-5 to -18 dB SNR (SSN)	80 monosyllabic words	4AFC tone identification	Tone 2 and Tone 3 are harder than Tone 1 and Tone 4
Lee 2013	25 in average	20	NH	0 to -15 dB SNR (SSN)	One monosyllabic word	4AFC tone identification	Tone 1 and Tone 2 are harder than Tone 3 and Tone 4

NH: normal hearing; 4AFC: 4-alternative forced-choice; 2AFC: 2-alternative forced-choice; dB SNR: decibel signal-to-noise ratio; SSN: speech spectrum noise; dB HL: decibel hearing level; SNHL: sensorineural hearing loss

Note: studies with prelingually deaf children wearing cochlear implants are not included in this summary. Binaural tone identification was assessed in all studies summarized in this table.

Despite being a frequently encountered paediatric group in clinical otology/audiology settings, children with OME-related hearing loss have not been investigated in terms of tone perception. Little is known about their possible difficulties with tone perception, especially when noise is present—as it is in typical classrooms [37–39]. Therefore the purpose of the present study was to describe monaural tone perception in school age children with OME-related hearing loss, to evaluate the interaction of noise, tone type, age, and hearing status on tone perception, and to assess the application of the hierarchical clustering method in profiling children with OME from the perspective of tone perception ability. The hypotheses were: (1) monaural tone perception in children with OME is poorer than children with NH; (2) monaural tone perception impairment in children with OME can be stratified by the hierarchical clustering algorithm based on pure tone hearing thresholds; and (3) hearing levels, background noise, age, and tone types have influence on monaural tone perception in children with OME-related hearing loss.

## Methods

### Ethical considerations

The Human Research Ethics Committee for Non-Clinical Faculties of the University of Hong Kong approved the study protocol (Reference No. EA430914). The study was also approved by the Ethics Committee of Shenzhen Children’s Hospital.

### Participants

School age Mandarin speaking children with a diagnosis of OME were sequentially recruited from the Department of Otorhinolaryngology-Head and Neck Surgery in Shenzhen Children’s Hospital, China. Most of the clinic attendances were due to parent-suspected hearing problems or routine follow-up after acute otitis media. Another group of Mandarin speaking school age children attending Shenzhen Children’s Hospital, but with normal hearing and normal middle ear function, participated as a control group. Written consents were obtained from parents or caregivers before data collection. Background and demographic information was provided by caregivers. A survey on present and past medical history was conducted before hearing tests, in the format of a questionnaire completed by parents or caregivers. All participants reported no history of preterm birth, craniofacial abnormalities, sensorineural hearing loss, chronic purulent otitis media, or middle ear surgeries. All children invited in the present study attended mainstream primary schools and were without known cognitive impairment.

### Procedures

Participants were examined by otoscopy, tympanometry, ipsilateral acoustic reflex, pure tone audiometry, and speech audiometry. Otoscopy was performed by the first author, a qualified otolaryngologist, using a portable otoscope (Welch-Allyn Inc., NY, USA). Indications for middle ear effusion included tympanic membrane retraction with a shorter malleus handle, absent or malformed reflective light cone, tympanic membrane discoloration, and visible air-fluid levels or bubbles [40].

Tympanometry was performed in a quiet room using a middle ear analyzer (TympStar, GSI, Eden Prairie, MN), calibrated to ANSI S3.39–1987 (R 2007) standards [41], with a continuous probe signal of 85 dB SPL at 226 Hz frequency and a sweep rate of 50 daPa/s. Recordings included the equivalent ear canal volume, peak compensated static acoustic admittance, tympanometric gradient, and tympanometric peak pressure. Type B and C2 tympanograms, categorized according to Jerger’s classification [42], were considered as indicators for OME.

The frequency tested for acoustic reflexes was 1000 Hz in an ipsilateral condition. Stimulation commenced at 90 dB HL, and then increments of 5 dB HL were given until a response was obtained or a maximum stimulation level of 105 dB was reached. An absent ipsilateral acoustic reflex was considered as evidence of OME.

A pure tone audiometer (204A, Entomed, Sweden) with insert earphones (ER-3A, Etymotic Research, Elk Grove Village, IL) was used to measure hearing thresholds of participants in a sound-treated booth. Background noise levels of the booth were measured by a sound level meter (type 2250, Brüel & Kjær, Nærum, Denmark). The background noise was within the maximum permissible ambient noise levels for pure tone audiometry with insert earphones [43]. Air conduction pure tone thresholds at 125 Hz, 250 Hz, 500 Hz, 1000 Hz, 2000 Hz, 4000 Hz, and 8000 Hz were tested. If any of the thresholds at frequencies from 250 Hz to 4000 Hz were greater than 20 dB HL, bone conduction thresholds from 250 Hz to 4000 Hz were obtained. The administration procedure followed the modified Hughson-Westlake method [44, 45]. The audiometer and headphone assembly was calibrated with a sound level meter (Type 824, Larson Davis, Depew, NY) according to standard specifications for audiometers [46].

Tone perception tests were performed in the same sound-treated booth using insert earphones. Speech stimuli were presented monaurally. For children with unilateral OME, the affected ears were tested. For children with bilateral OME, right ears or left ears were randomly assigned for testing.

The lexical tone subtest of the Mandarin Pediatric Lexical Tone and Disyllabic-Word Picture Identification Test in Noise (MAPPID-N) was used to measure tone perception in children with age above four years [47]. MAPPID-N is a closed-set computerized picture identification test. In the lexical tone subtest, there are a total of six sets of monosyllabic lexical tone test items. Each test set has four items, which represent four different lexical tones. Children need to choose from four pictures in a two-rows-two-columns format. Therefore, a total of 24 items were tested. At adverse listening conditions, children were encouraged to guess the most likely answer. In this type of task, where listening takes place in a sometimes ambiguous situation, participants may make decisions based on non-sensory factors rather than on true perceptual estimation [48]. Considering the potential for this type of response bias, children were not forced to choose an answer. They could refrain from responding if they failed to identify a tone. Participants were tested monaurally. Both speech and noise were presented to the tested ear. Speech spectrum noise was used and the root-mean-squared intensity was calibrated to 65 dBA. The intensity of tone stimuli varied to achieve different SNRs. The test was conducted first in quiet to familiarize children with the test items. Tone recognition score, which was the percentage of correct answers under each SNR, was recorded automatically by the MAPPID-N software. If the score was less than 70%, the test was repeated until a score of 70% was reached. Secondly, monosyllabic tone stimuli and noise were presented under a series of SNRs to obtain a tone recognition threshold. -9 dB SNR was chosen initially. If the score was above 80%, then the SNR was reduced by 3 dB and the test repeated until the score fell below 20%. If the initial -9 dB SNR achieved scores less than 80%, then the test was repeated with SNR increased by 3 dB steps until a score of at least 80% was reached. Tone recognition score for each lexical tone, which was the percentage of correct answers under each SNR for Tone 1, Tone 2, Tone 3, or Tone 4, was also recorded for analysis. From -9 dB SNR to -21 dB SNR, the overall root-mean-squared presentation level of test stimuli ranged from 65.64 dB SPL to 65.03 dB SPL. The presentation sequence was not randomized, in order to avoid possible learning effects which may exaggerate the improved performance demonstrated under better listening conditions. The presentation sequence took 20 minutes to complete.

### Statistical analysis

Tone recognition threshold was defined as the SNR under which the child had a 50% correct tone recognition score and was calculated using mathematical procedures similar to Nissen et al. [49]. Logistic regression, as shown in Eq (1), was used to obtain the regression slope (*b*) and regression intercept (*a*) based on the proportion correct score (*p*) at each SNR for each participant.

$$\text{log}\frac{p}{1-p}=a+b\times SNR$$ (1)

In order to obtain the tone recognition threshold, which was the SNR at which *p* was estimated to be 50%, Eq (1) can be solved to Eq (2) and then simplified to Eq (3) to generate a SNR for a 50% correct score as *SNR* (50%), using the regression slope and regression intercept values obtained from Eq (1).

$$\text{log}\frac{50\%}{1-50\%}=a+b\times SNR\left(50\%\right)$$ (2)

$$SNR\left(50\%\right)=-\frac{a}{b}$$ (3)

Correlation analysis was used to examine the relationship between tone recognition threshold and PTA and chronological age. T-test and one-way ANCOVA were used to investigate the effect of hearing status on tone perception. A mixed design repeated-measures ANCOVA was used to investigate the effects of listening condition, tone type, and hearing status on tone perception. The statistically significant level was set at 0.05 for main comparisons and the critical value for significance for *post hoc* analysis was Bonferroni corrected [50].

## Results

Ninety-six children who were examined and diagnosed with OME between December 2014 and August 2015 were invited to participate in the present study. Forty-nine children with normal middle ear function and NH were recruited as a control group. Children with tone recognition thresholds which were beyond 2 standard deviations of the mean value were excluded from the final analysis. The averaged pure tone thresholds in two participants were beyond the speech presentation level at the most adverse listening condition tested (-18 dB SNR), which made their tone recognition perception results unreliable. These two participants were also excluded. Forty-one children with NH and 84 children with OME were included in the ultimate data analysis. The age range of the 41 children with NH was from 73 months to 166 months, with a mean age of 103 months. The age range of the 82 participants with OME was from 72 months to 144 months, with a mean age of 96 months. Linear correlation analysis was performed for possible age effects among children with NH and children with OME, for tone recognition threshold. For children in the control group, there was no significant correlation between age and tone recognition threshold, *p* = 0.504. However, tone recognition threshold in children with OME was significantly correlated with age, *p* = 0.001, *r* = -0.346. Therefore age was considered as a covariate in the following analyses.

Linear correlation analysis was also carried out to investigate whether tone perception in children with OME was related with PTA results. Pure tone thresholds at frequencies from 125 Hz to 8000 Hz showed significant correlation with tone recognition threshold, with *p* < 0.01 at all frequencies and moderate correlation coefficient *r* ranges from 0.32 to 0.43.The most prominent correlation occurred between tone recognition threshold and PTA at 500 Hz. No significant correlation existed between age and PTA results in children with OME, *ps* > 0.05.

**Fig 2:**
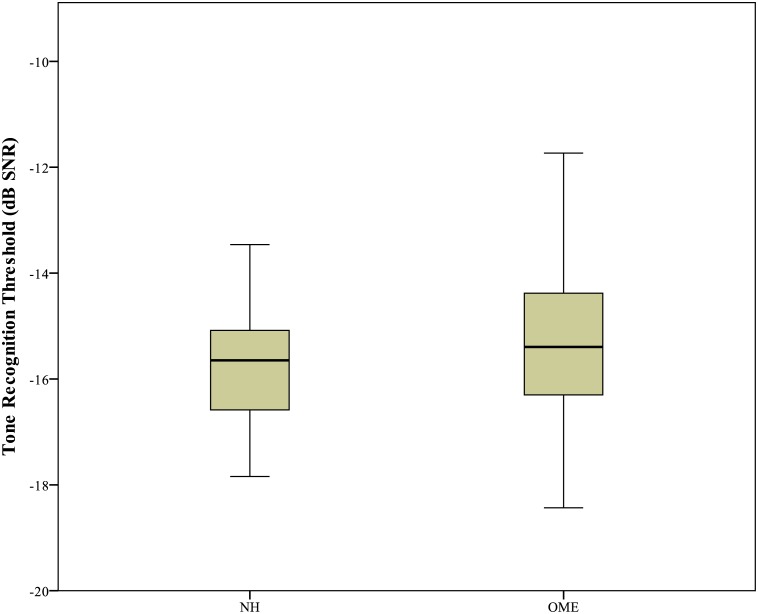
Boxplot of tone recognition thresholds in children with NH and in children with OME.

**Fig 3:**
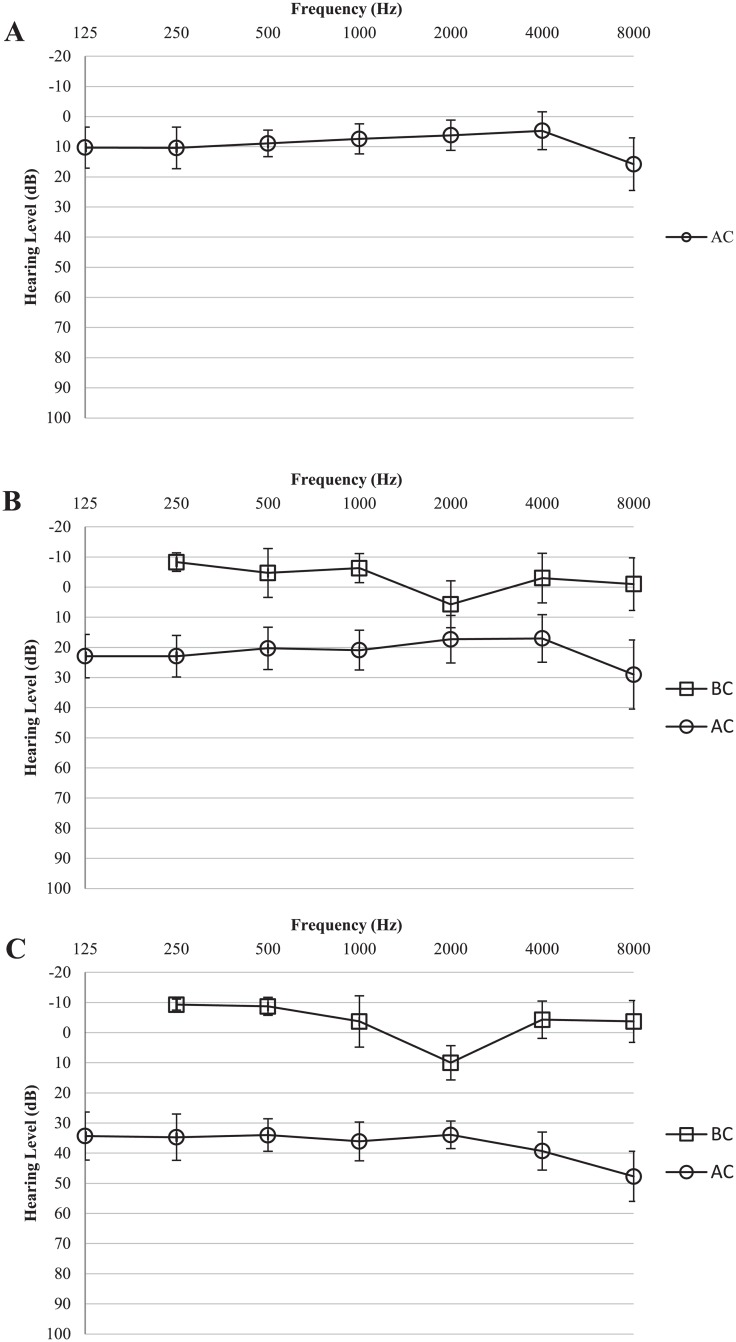
Mean audiogram based on the mean thresholds and SDs at all frequencies, for participants in each group. A. Mean audiogram of children with NH. B. Mean audiogram of children in the OME-A group. C. Mean audiogram of children in the OME-B group.

**Fig 4:**
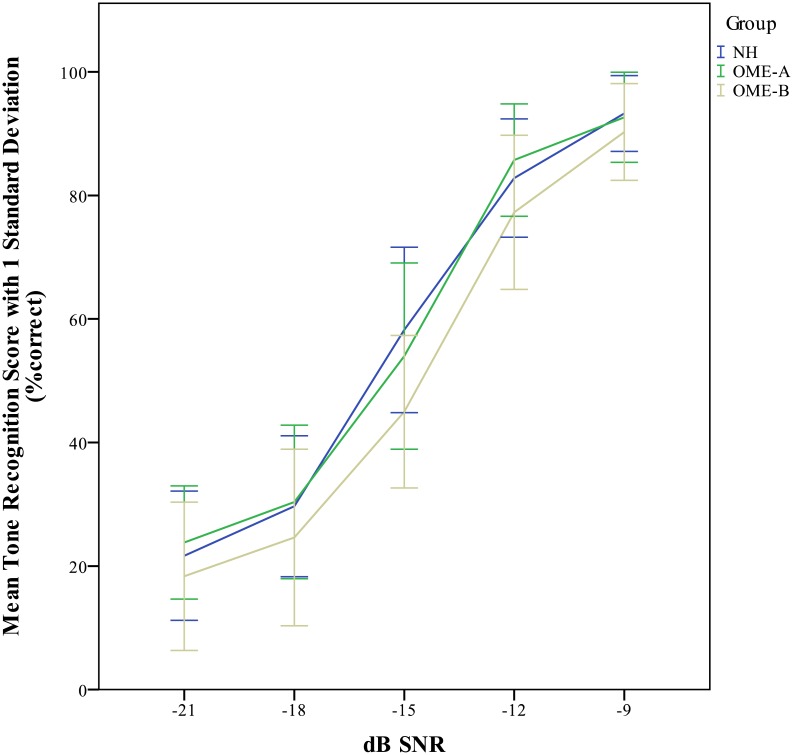
Mean tone recognition scores and SDs at different SNRs in each group.

On average, participants with normal hearing demonstrated better performance in tone perception (*M* = -15.82, *SE* = 1.05), than those with OME (*M* = -15.39, *SE* = 1.40). The difference, -0.43, was not significant, *t* (21) = -1.734, *p* = 0.085.The distribution of tone recognition thresholds in children with NH and in children with OME is displayed in Fig 2. Children with OME showed a wider range of tone recognition threshold compare to their NH peers. The insignificant difference between children with OME and children with NH based on group means may mask any relatively large individual variation within the group of children with OME. Subgroups were therefore created to better examine tone recognition difficulties in children with OME, especially in those with poorer hearing thresholds. Children with OME were stratified into two groups using the hierarchical cluster algorithm approach previously described [26]. Based on the previous methodology, participants categorised into Clusters 1 and 2 were pooled together as OME-A, and children categorised into Clusters 3 and 4 were pooled together as OME-B for further analysis. Table 2 shows the group average PTA and tone recognition thresholds for the two groups of children with OME and for the control group of children with NH. Fig 3 shows the group mean audiogram for each group. Fig 4 displays the mean tone recognition scores of children in the three groups at each SNR.

**Table 2:** Mean PTA and tone recognition threshold in each group.

Group	Age, M ± SD (months)	Pure tone threshold, M ± SD (dB HL)							Tone recognition threshold, M ± SD (dB SNR)
		125Hz	250Hz	500Hz	1000Hz	2000Hz	4000Hz	8000Hz	
NH (n = 41)	103.2 ± 21.9	10.3 ± 6.8	10.4 ± 6.9	8.9 ± 4.4	7.4 ± 5.0	6.2 ± 5.0	4.7 ± 6.3	15.8 ± 8.7	-15.8 ± 1.1
OME-A (n = 47)	98.6 ± 19.7	22.9 ± 7.2	22.9 ± 6.9	20.3 ± 7.0	20.9 ± 6.6	17.3 ± 7.9	17.0± 7.9	29.0 ± 11.5	-15.8 ± 1.2
OME-B (n = 35)	91.7 ± 17.7	34.3 ± 8.0	34.7 ± 7.7	34.0 ± 5.4	36.1 ± 6.4	33.9 ± 4.6	39.3 ± 6.3	47.7 ±8.3	-14.7 ± 1.4

M: mean; SD: standard deviation; dB SNR: decibel signal-to-noise ratio; dB HL: decibel hearing level

One-way ANCOVA was conducted to investigate the effect of hearing status on tone recognition thresholds with age as the covariate. It was found that the covariate, age, was significantly related to the tone recognition threshold, *F*(1, 119) = 7.273, *p* = 0.008, partial *η*^*2*^ = 0.058. There was also a significant effect of hearing status on tone recognition thresholds after controlling for the effect of age, *F*(2, 119) = 6.212, *p* = 0.003, partial *η*^*2*^ = 0.095. Pairwise comparison revealed that OME-B group children demonstrated significantly poorer tone recognition thresholds compared to children with NH, *t*(119) = -2.918, *p* = 0.004, partial *η*^*2*^ = 0.067, and compared to OME-A group children, *t*(119) = -3.284, *p* = 0.001, partial *η*^*2*^ = 0.083. However, there was no significant difference in tone recognition threshold between children with NH and children in the OME-A group, with *p* = 0.993.

In order to investigate the effects of listening condition, tone type, age, and hearing status on tone perception, a mixed-design repeated-measure ANCOVA was used with different SNRs and tone types (Tone 1, Tone 2, Tone 3, and Tone 4) as the within-subject variables, different groups as the between-subject variable, age as the covariate, and tone recognition score for each lexical tone at each SNR as the dependent variable. Since tone recognition scores for each lexical tone at -9 dB SNR and -21 dB SNR were influenced by ceiling and floor effects, respectively, they were excluded from this analysis. Thus, tone recognition scores for each lexical tone obtained at -12, -15, and -18 dB SNR were entered into the analysis. The results are displayed in Table 3.

**Table 3:** Results from the mixed-design repeated-measure ANCOVA.

Factor		Statistical test	Test statistic	Significance	Effect size
Listening condition	Main effect	Within-subject effect	*F*(2, 216) = 29.207	0.000^a^	Partial *η*^*2*^ = 0.213
	-12 *vs* -15 dB SNR	Within-subject contrast	*F*(1, 108) = 21.325	0.000^a^	Partial *η*^*2*^ = 0.165
	-15 *vs* -18 dB SNR		*F*(1, 108) = 11.361	0.001^a^	Partial *η*^*2*^ = 0.095
Tone type	Main effect	Within-subject effect	*F*(2.764, 298.56) = 2.805	0.044^a^	Partial *η*^*2*^ = 0.025
	Tone 1 *vs* Tone 2	Pairwise comparison	Mean difference = 0.884	0.000^b^	
	Tone 1 *vs* Tone 3		Mean difference = 1.252	0.000^b^	
	Tone 1 *vs* Tone 4		Mean difference = 0.918	0.000^b^	
	Tone 2 *vs* Tone 3		Mean difference = 0.367	0.005^b^	
	Tone 2 *vs* Tone 4		Mean difference = 0.034	1.000	
	Tone 3 *vs* Tone 4		Mean difference = -0.333	0.002^b^	
Hearing status	Main effect	Between-subject effect	*F*(2, 108) = 6.498	0.002^a^	Partial *η*^*2*^ = 0.107
	OME-A *vs* Control	Pairwise comparison	Mean difference = 0.042	1.000	
	OME-B *vs* Control		Mean difference = -0.365	0.013^b^	
	OME-A *vs* OME-B		Mean difference = 0.407	0.003^b^	
Interaction between tone type and listening condition	Main interaction effect		*F*(6, 648) = 1.153	0.330	
Interaction between listening condition and hearing status	Main interaction effect		*F*(4, 216) = 1.020	0.398	
Interaction between tone type and hearing status	Main interaction effect		*F*(5.529, 298.56) = 3.117	0.007^a^	Partial *η*^*2*^ = 0.055

^a^*p* < 0.05 with Bonferroni correction.

^b^*p* < 0.05 with Bonferroni correction.

Note: Mauchly’s test indicated that the assumption of sphericity had been violated for the main effects of tone type, *χ*^*2*^(5) = 13.993, *p* = 0.016. Therefore degrees of freedom were corrected using Greenhouse-Geisser estimates of sphericity for the main effect of tone type, and the interaction between tone type and hearing status.

There was a significant main effect of listening condition on tone recognition. The more adverse the SNR, the poorer tone performance participants demonstrated—for all groups and tone types. Different tones had different tone recognition scores. Recognition of Tone 1 was significantly better than recognition of other tones, with all comparisons *p* < 0.000, and the recognition of Tone 3 was significantly worse than that of other tones. In other words, Tone 1 was the easiest tone to identify and Tone 3 was the most difficult tone to identify for all participants. The significant effect of hearing status on tone recognition was primarily contributed by the children in the OME-B group, due to the significant difference in tone recognition score between children in the OME-A group and children in the OME-B group, and between children in the control group and children in the OME-B group, while tone recognition was not significantly different between children with normal hearing and children in the OME-A group.

There was no significant interaction effect between listening condition and tone type, which indicated that the recognition of all tones decreased as the listening condition became more adverse. There was no significant interaction effect between listening condition and hearing status on tone recognition score. This indicated that children with or without hearing loss showed similar decreased tone recognition when listening condition deteriorates. In other words, noise played a much more important role in tone perception than hearing status or tone type, bearing in mind the large effect size of listening condition (0.213) and moderate effect size of hearing status (0.107) and small effect size of tone type (0.025) when considered separately.

The significant interaction effect between tone type and hearing status indicated that the recognition score for different tones differed among three groups. To explore this interaction, four repeated-measures ANCOVAs were conducted with listening condition as the within-subject variable, hearing status as the between-subject variable, and tone recognition score for each lexical tone as the dependent variable. The results are displayed in Table 4.

**Table 4:** Results of four repeated-measure ANCOVAs.

Test item		Statistical test	Test statistic	Significance, *p*	Effect size
Tone recognition score for Tone 1	Main effect	Between-subject effect	*F*(2, 108) = 6.177	0.003^a^	Partial *η*^*2*^ = 0.103
	OME-A *vs* Control	Pairwise comparison	Mean difference = 0.305	0.266	
	OME-B *vs* Control		Mean difference = -0.361	0.218	
	OME-A *vs* OME-B		Mean difference = 0.666	0.002^b^	
Tone recognition score for Tone 2	Main effect	Between-subject effect	*F*(2, 109) = 7.590	0.001^a^	Partial *η*^*2*^ = 0.122
	OME-A *vs* Control	Pairwise comparison	Mean difference = 0.313	0.213	
	OME-B *vs* Control		Mean difference = - 0.396	0.122	
	OME-A *vs* OME-B		Mean difference = 0.708	0.001^b^	
Tone recognition score for Tone 3	Main effect	Between-subject effect	*F*(2, 109) = 2.405	0.095	
Tone recognition score for Tone 4	Main effect	Between-subject effect	*F*(2, 109) = 1.630	0.201	

^a^*p* < 0.05with Bonferroni correction.

^b^*p* < 0.05 with Bonferroni correction.

These four separate repeated-measure ANCOVAs indicated that recognition for Tone 1 and Tone 2 decreases as hearing ability decreases, and recognition for Tone 3 and Tone 4 does not change significantly with hearing acuity levels. This result suggests that the noted differences in tone recognition thresholds were mainly due to differences in Tone 1 and Tone 2 recognition scores.

**Fig 5:**
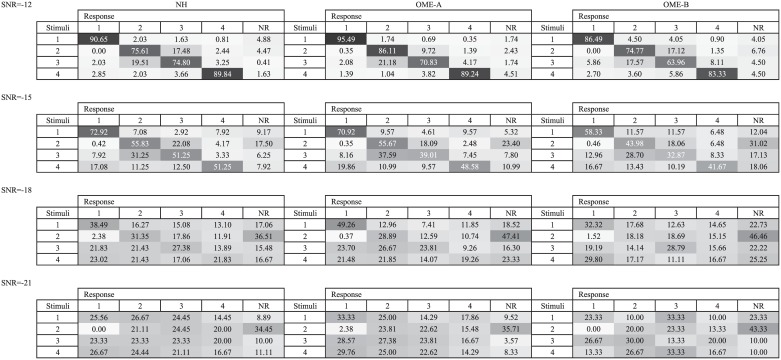
Tone recognition confusion matrices of three child groups under -12 dB SNR to -21 dB SNR. Data were pooled from all participants in each group. For each panel of 4 × 6 cells, the rows indicate the stimuli and the columns indicate the response tone types. The grey scale in each cell and the value in it represent percentage of responses. NR: no response.

Fig 5 displays the tone recognition confusion matrices for the three groups. It can be visualized that confusions most frequently occur between Tone 2 and Tone 3. However, as listening condition worsens, such as at -18 and -21 dB SNRs, the error pattern is more or less evenly distributed among Tone 2, Tone 3 and Tone 4, while Tone 1 remains the easiest tone to recognize.

## Discussion

### Effects of OME on tone perception

To the authors’ knowledge the present study is the first attempt to report on tone perception in children with OME. Grouping based on hierarchical cluster analysis demonstrated that meaningful stratification in tone perception results could be achieved in children diagnosed with OME. Children in the OME-A group showed similar tone recognition performance to children with NH, and significantly better performance than children in the group with greater degrees of OME-related hearing loss. Tone recognition threshold was found to be correlated with pure tone hearing threshold at all frequencies, especially with PTA threshold at 500 Hz. Similar correlation was also reported in earlier studies investigating Mandarin tone perception in quiet environments in adults with sensorineural hearing loss [17, 21] and studies investigating tone perception in noise in a paediatric group with moderate to profound sensorineural hearing loss [51]. Previous studies indicated that low frequency hearing threshold may be more closely related with tone perception [16, 17]. However, since the pure tone audiogram configuration of participants in the present study was relatively flat in shape, no significant difference was identified in the correlation coefficients between tone perception ability and hearing thresholds at the low or high frequency range.

### Effects of tone type on tone perception

Tone type was a significant factor for tone recognition performance. The four lexical tones in Mandarin demonstrated different levels of difficulty. Tone 1 was the easiest to identify while Tone 3 was the hardest to recognize for all participants in the current study. Similarly, Tone 1 was also reported to be the easiest and Tone 3 as the most difficult tone in an adult population by Krenmayr et al. [35] and in a paediatric group by Mao and Xu [20]. Tone 3 is also the last tone to achieve mature perception in Mandarin-speaking children with normal hearing [33]. Matrix analysis indicated that Tone 2 and Tone 3 were the most confusing tones. This finding agrees with earlier studies in children with normal hearing in both a noisy environment [20] and a quiet environment [34] and in a quiet environment for children with profound sensorineural hearing loss [22]. Tone 2 is a high rising tone and Tone 3 is a low dipping tone with a concave contour. However, the contour change in Tone 3 may be misperceived and result in the identification of a low rising tone similar to that of Tone 2 in the spectral domain [34]. Tone 2 usually has a slight dip at the 20% duration point of the vowel and the contour change of Tone 3 typically occurs at the 50% duration point of the vowel. In daily speech, the rising part of Tone 2 may appear later and become similar to that of Tone 3 in the temporal domain [52]. Therefore, the similarities in both the F0 temporal and spectral domains shared by Tone 2 and Tone 3 may lead to the high confusion rate found between the two tones (see Fig 1).

The interaction between tone type and hearing status revealed that tone recognition of Tone 1 and Tone 2 were differed significantly among the three listener groups and that Tone 3 and Tone 4 recognition abilities did not differ significantly. Easier tones (Tones 1) are more affected by the hearing threshold status of children than more difficult tones (Tones 3). Children with OME-related hearing loss demonstrated more impaired tone perception for the tones that were, overall, found to be relatively easier. For relatively more difficult tones, tone perception performance was more or less equally poor among all children with or without hearing loss. It needs to be noted that the listening conditions under which the difference in recognition between relatively easier and harder tones occurred were very adverse and may not reflect the situation under real world listening environments. In addition, the effect size of this interaction was small to moderate (partial *η*^*2*^ = 0.055), indicating significant but weak interaction effects.

### Effects of listening condition on tone perception

The present study showed that listening condition affected tone recognition in children with or without OME. This detrimental effect appears earlier and is more prominent in children with greater levels of OME-related hearing loss. At -9 dB SNR, tone recognition performance is similar in all three groups of participants. However, at listening conditions which are more adverse than -9 dB SNR, tone recognition performance in OME-B group children degrades more rapidly than for their peers with normal hearing or minor hearing loss. Previous studies on classroom acoustics showed that the background noise in many primary schools may be more adverse than -9 dB SNR [37, 53]. Considering that monosyllabic lexical tones in a picture identification task are much easier than words or sentences to recognize under the same listening environment, it is not unreasonable to conclude that children with OME are negatively affected by typical classroom noise to a larger extent than children with normal hearing.

### Age and other effects on tone perception

In the present study, children with normal hearing demonstrated near perfect tone recognition scores at -9 dB SNR with 96.3% correct answers. The result agrees with findings reported from a group of children with normal hearing [31] and findings observed from two adult groups with normal hearing [35, 36]. There was no developmental change for tone perception in noise in children with NH in the present study. Similar findings were also reported by Yuen and Yuan [30], and Zhu [54]. However, chronological age was significantly correlated with tone perception in noise in children with OME. Older children with OME performed better than younger children with OME. One possible reason is that cognitive load is activated or employed for this task as compensation for hearing impairment. Extra cognitive involvement is not necessary for tone recognition in noise in children with NH. However, increased cognitive load is required when children have hearing loss. Therefore older children, with more developed cognitive reserve or mobilization function, outperform younger children with OME. Another possible reason is listening effort. Several studies demonstrated that children with mild to moderate sensorineural hearing loss expended more listening effort under noisy environments [55, 56]. It is possible that the noisier the listening environment is, the more listening effort is required, and that increasing chronological age reduces the degree of effort expended. One clinical implication for the interaction of age and hearing impairment on tone perception in noise is that younger children with OME may justifiably be given priority in hearing intervention and rehabilitation owing to the more adverse impact of their hearing loss on tone perception compared with older children.

The disparity in tone performance demonstrated using different tests may reflect the fact that tone perception is affected by testing format and test material to a large extent. Lui et al. [9] reported that compared to a conventional four-alternative forced-choice paradigm, tone perception which was indicated by repetition of tone and judged by test raters was scored much higher. In tests using a four-alternative forced-choice paradigm, the cognitive load associated with assigning tones to different categories may affect tone perception. In addition, representativeness and phonetic structure of test material and response bias may also contribute to the disparate conclusions of different studies [35]. Therefore a standardized lexical tone test would be useful to expand knowledge in this area.

In the present study, tone identification was tested monaurally rather than binaurally. Binaural hearing in real life settings is more complex than monaurally tested tone perception. Research indicates that listeners with NH obtain more binaural gain than listeners with hearing loss in noisy environments [57]. There is also evidence that asymmetric hearing loss creates extra speech perception difficulties for children, especially under noise [58–60]. In future studies, binaurally tested tone perception is needed to more realistically reflect the effects of listening environments on children with OME-related hearing loss.

## Conclusions

A hierarchical clustering algorithm was used to create a meaningful stratification of hearing impairment in children with OME-related hearing loss. Tone perception in noise in school age children with a greater level of OME-related hearing loss was suboptimal compared to children with NH. This group of children also had more impaired perception for Tone 1 and Tone 2 compared to their peers with lesser degrees of OME-related hearing loss, while perception of Tone 3 and Tone 4 remained similar among the three groups. It was found that Tone 3 was more difficult to identify while Tone 1 was easier to identify than other tones. Tone perception in noise in younger children with OME is more influenced by OME related hearing loss than that in older children.
